# Breast implant associated EBV-positive Diffuse Large B-cell lymphoma: an underrecognized entity?

**DOI:** 10.1186/s13000-023-01337-5

**Published:** 2023-04-25

**Authors:** Johanna Vets, Lukas Marcelis, Charlotte Schepers, Yaliva Dorreman, Sanne Verbeek, Lieve Vanwalleghem, Katrien Gieraerts, Liesbeth Meylaerts, Jan Lesaffer, Helena Devos, Natalie Put, Sylvia Snauwaert, Pascale De Paepe, Thomas Tousseyn

**Affiliations:** 1 Translational Cell and Tissue Research Laboratory, KU Leuven, Leuven, Belgium; 2 Department of Pathology, UZ Leuven, University Hospitals, Leuven, Belgium; 3 Department of Oncological Surgery, UZ Ghent, Brugge, Belgium; 4 Department of Pathology, ZOL, Genk, Belgium; 5 Department of Pathology, AZ Sint-Jan, Brugge, Belgium; 6 Department of Radiology, AZ Sint-Jan, Brugge, Belgium; 7 Department of Radiology, ZOL, Genk, Belgium; 8 Department of Oncological Surgery, Sint-Jan, Brugge, AZ Belgium; 9 Department of Laboratory Medicine, AZ Sint-Jan, Brugge, Belgium; 10 Department of Hematology, ZOL, Genk, Belgium; 11 Department of Hematology, AZ Sint-Jan, Brugge, Belgium

**Keywords:** EBV + DLBCL, Breast implant associated lymphoma, BIA-DLBCL, FA-DLBCL, CI-DLBCL

## Abstract

Breast-implant associated (BIA) lymphoma is an infrequent type of cancer occurring in the fluid and fibrous capsule around a textured breast implant. Recently, both the 2022 WHO 5th edition classification of Haematological tumours (WHO HAEM5) and 2022 International Consensus Classification of Mature Lymphoid Neoplasms (22ICC), recognized breast implant-associated Anaplastic Large Cell Lymphoma (BIA-ALCL) as a definitive entity, defined as a mature CD30-positive T-cell lymphoma, confined by a fibrous capsule, in a breast implant setting. Only few B-cell lymphomas have been reported in the literature to be associated with breast implants. Here we report two EBV-positive Diffuse Large B-cell lymphomas (EBV + DLBCL) in relation to a breast implant, both expressing CD30 as well as EBV latency type 3. Both lesions were considered as DLBCL associated with chronic inflammation (CI-DLBCL), but one presented as a 7 cm solid mass, while the other presented as a fibrin-associated DLBCL (FA-DLBCL) in an HIV patient. Clinically, both are in complete remission 6 months or longer after capsulectomy and graft removal, without additional chemotherapy.

Such cases, characterized by large CD30-positive cells, can easily be misdiagnosed as BIA-ALCL if the cell of origin is not further established. Therefore, a diagnostic panel including lineage-specific B-and T-cell markers and EBER in situ hybridization is essential to recognize this rare entity, to understand lymphomagenesis, to predict outcome and to define clinical approach.

## Introduction

Breast implant associated lymphomas are infrequent complications of reconstructive prosthetic breast surgery. Most BIA lymphomas are of T-cell origin, and are classified as anaplastic lymphoma kinase negative (ALK-)ALCL. The entity BIA-ALCL has recently been recognized in the 22ICC and WHO HAEM5 as a distinct clinicopathological entity [1, 2]. Only occasional reports of BIA-large B-cell lymphomas have been published so far. The clinical and pathological characteristics and treatment modalities are therefore less defined. We report two cases of BIA-large B-cell lymphomas and review the literature, illustrating the clinical and morphological spectrum of these lesions.

## Case presentation

### Case 1

**Fig. 1:**
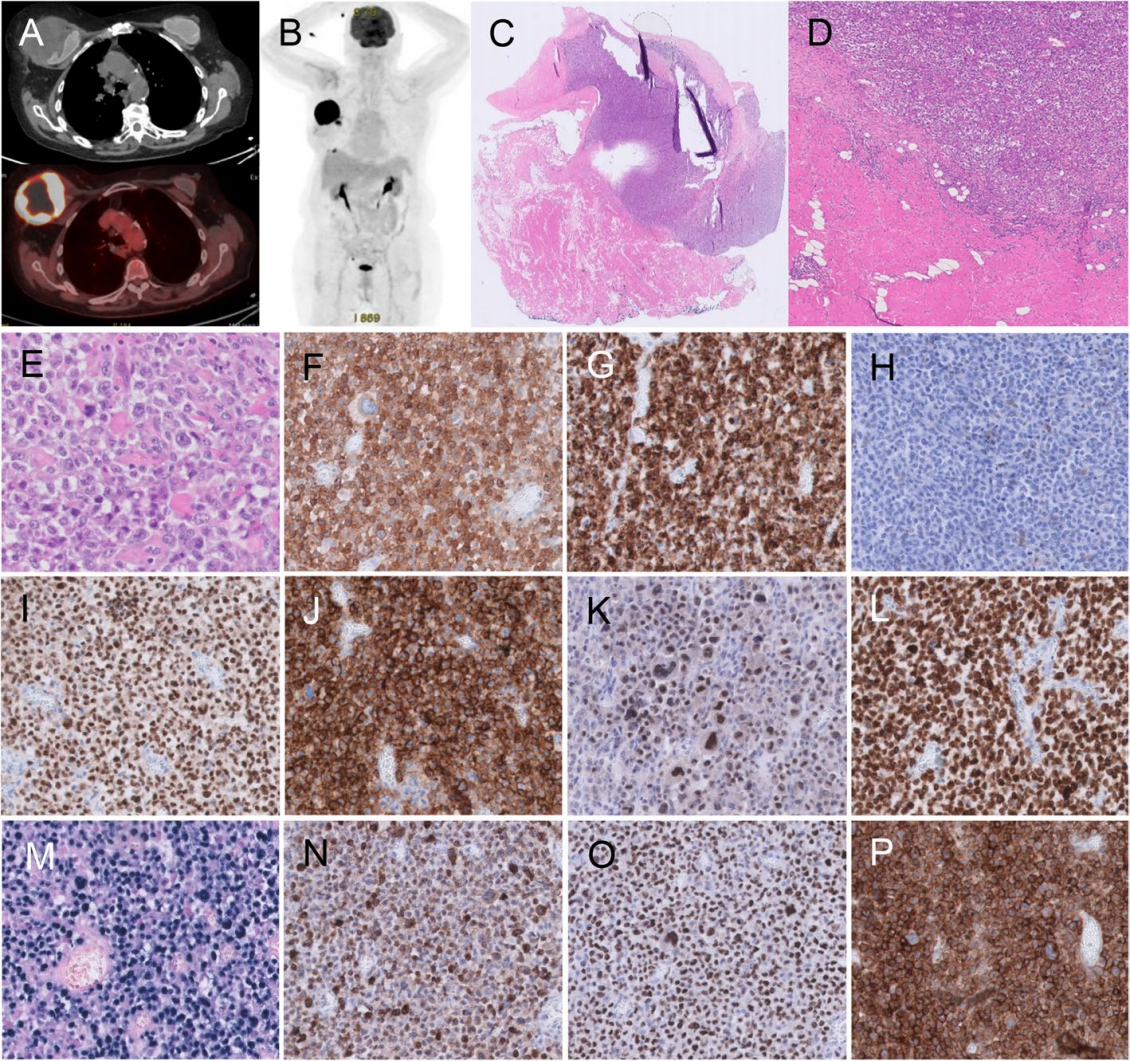
Case 1. **A**-**B**. PET/CT shows a large tumoral mass at the upper-external quadrant of the right breast, in close contact with the implant with a layer of fluid surrounding the implant; There were no lymphadenopathies nor other suspicious PET-positive lesions. **C**-**D**. Low power view of the H&E of the tumor mass, composed of diffuse sheets of large atypical lymphoid cells. **E**. High power field view of HE of large malignant cells expressing CD30 (**F**), CD15 (**G**), but no T-cell markers, such as CD4 (**H**). The cells did express B-cell markers PAX5 (**I**), CD20 (**J**), OCT2 (**K**), CD79A (**L**) and were positive for EBER (**M**); The expression of LMP1 (**N**) and EBNA2 (**O**) indicate EBV viral latency type III; The tumor microenvironment is nearly absent, but the tumor cells diffusely express PD-L (**P**)

A 75-year old female developed hypogammaglobulinemia as a consequence of chronic steroid treatment for Chronic Obstructive Pulmonary Disease (COPD). Twenty-seven years ago, the patient had a left-sided mastectomy with adjuvant hormonal therapy for an invasive breast carcinoma of no special type (NST), followed by bilateral (textured) prosthetic reconstruction. After nineteen years, bilateral capsulectomy and prosthesis replacement was performed because of bilateral Baker grade IV capsular contracture and leakage. Capsulectomy specimen was radiologically and histologically assessed confirming implant rupture. Eight years later she presented with a rapidly growing palpable mass in the right breast. She reported a 5 kg weight loss but no other B-symptoms. Clinical examination and imaging studies (ultrasound, MRI and PET-CT) confirmed a tumoral mass at the upper-external quadrant of the right breast, in close contact with the implant with a layer of fluid surrounding the implant (see Fig. 1A-B). There were no lymphadenopathies nor other suspicious PET-positive lesions. LDH level was highly elevated (587 U/L) and there was mild inflammation (CRP 25.8 mg/L). An EBV viral load of 14,747 IU/ml was detected in the peripheral blood.

Cytological examination of the peri-prosthetic fluid identified the presence of an atypical lymphoid cell population, that could not be observed in a cytological specimen of the right axillary lymph node. A core needle biopsy (CNB) of the mass was performed under ultrasound-guidance, which was partially necrotic and showed a diffuse infiltrate of large, atypical cells Apoptotic cells and mitotic figures were frequently observed but no diagnostic hallmark cells were identified. The large atypical cells strongly expressed CD30, CD20 and were positive on EBER in situ hybridization (EBER ish), and CD3 was only expressed in small reactive T-cells. These findings eventually led to a diagnosis of an EBV + DLBCL. Subsequently, a radical mastectomy, type Halsted, was performed, including the removal of the tumoral mass, the breast implant and its surrounding capsule, that was tightly attached to the chest wall. Consequentially, the major pectoral muscle was resected as well. On macroscopic examination revealed a pale yellow-white mass of approximately 7 cm. The mass was located within and tightly associated to the fibrotic breast capsule. Microscopic examination revealed a fairly circumscribed large lymphoid mass located between the collagenous strands of the capsule, but with only limited expansion into the surrounding striated muscle fibers (see Fig. 1C-D). The lymphoid cells were large with a nuclear size that was 3–5 × as large as nuclei of nearby endothelial cells, and their cytomorphological features were similar as described for the CNB (see Fig. 1E). The atypical cells expressed CD30 (see Fig. 1F), a complete B-cell phenotype (CD20 + , PAX5 + , OCT2 + , BOB1 + , CD79A +) and EBER (see Fig. 1I-M) but no CD3. Furthermore, the lymphoma was of non-germinal center origin (CD10-, BCL6 partially and weakly + , MUM1 +) and expressed BCL2 + and GATA3. The Ki-67 proliferation index was high (70–80%) and C-MYC was positive in approximately 30% of the atypical cells. There was no expression of the hypoxia marker Carbonic Anhydrase 9 (CA-9) in the tumor cells. The atypical cells showed an EBV latency type 3 (LMP1 + , EBNA2 +) and overexpressed PD-L1 (22C3) (see Fig. 1N-P). Multiplex PCR-based immunoglobulin (Ig) clonality testing revealed a clonal peak within a polyclonal background for the Ig Kappa target, whereas no fragments for Ig Heavy Chain were detected. Fluorescent In Situ Hybridization analysis showed absence of translocations in t(18q21)/*BCL2*, t(3q27)/*BCL6* and t(8q24)/*CMYC,* while gain of 9p24 containing the *PD-L1* gene was detected. Furthermore, massive parallel sequencing of a gene panel, including 51 genes important in lymphomagenesis, revealed two pathogenic variants: one in the NFKBIE gene, a c.137del p.(Ile46Thrfs*3) at 41% variant allele frequency (VAF) and a small clone in the DDX3X gene, c.1171-1G > A p.?, at 2.3% VAF. Furthermore a variant of unknown significance (VUS) was detected in the EZH2 gene, c.1391G > A p.(Gly464Glu), at 2.8% VAF.

Given the history of long-present breast implants with a history of implant rupture the final diagnosis of Diffuse large B-cell lymphoma (DLBCL) associated with chronic inflammation (CI-DLBCL) was made in concordance with the WHO HAEM5 (ICD-O 9680/3) and the 22ICC classifications. Currently, there are no staging guidelines for this disease, however, according to the available TNM staging for BIA-ALCL, this case would have a pT4N0M0 stage IIa disease, given the mass formation and infiltration beyond the implant capsule [3]. No bone marrow trephine was performed.

Considering the patient had an ECOG performance status of 2 and the explicit wish of the patient no additional (chemo)therapy was given. Follow-up FDG PET-scan showed complete metabolic remission 6 months post-surgery. The contralateral implant was left in place.

### Case 2

**Fig. 2:**
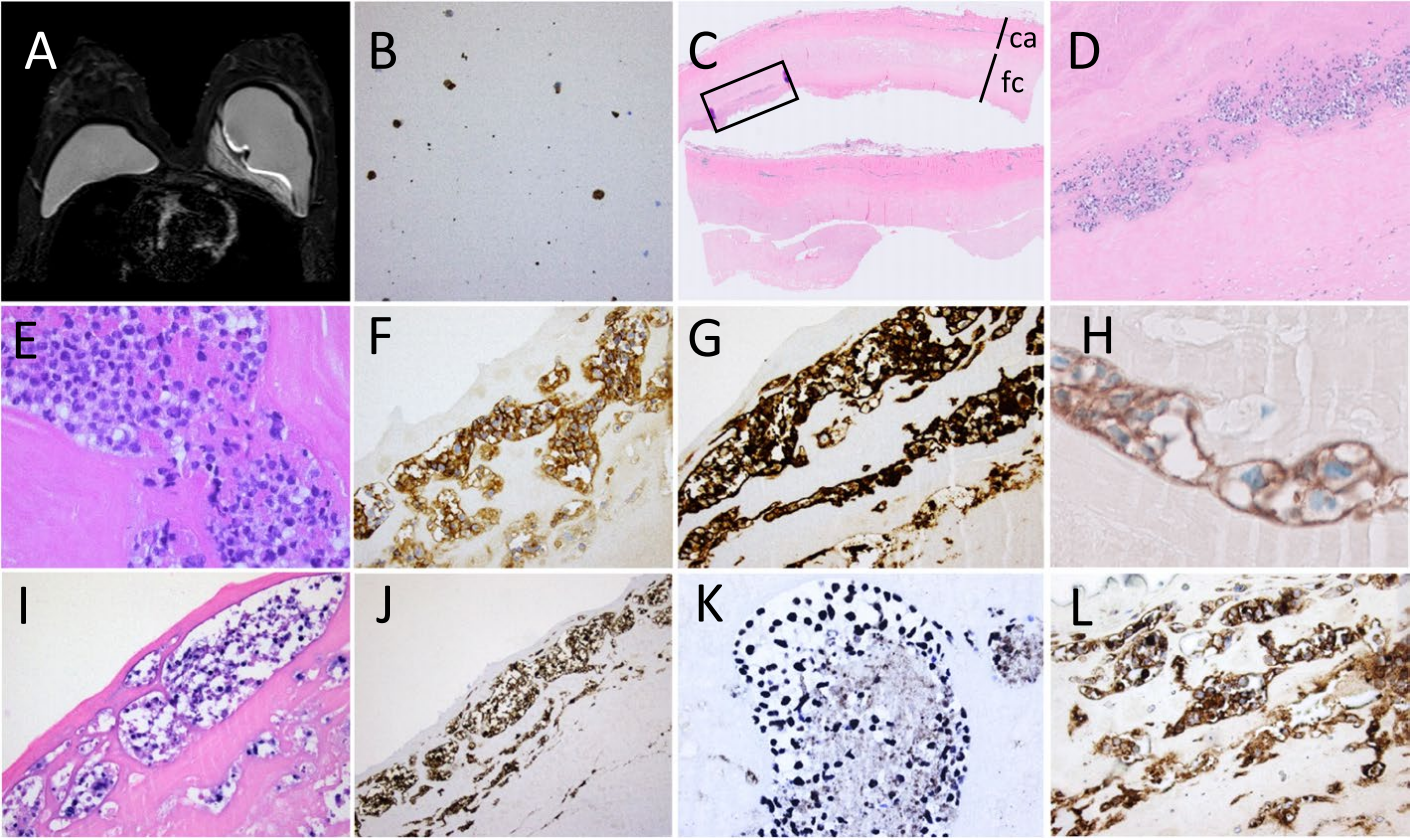
Case 2. **A**. MRI shows intact breast implants, and a thick-walled fluid collection with intralesional septations, but no contrast capturing, medially of the implant in the left breast; **B**. FNAC cytology shows large atypical cells (Papanicolaou stain), expressing CD30 and PAX5 (not shown); **C**. HE stain of the left capsulectomy specimen, showing a fibrous capsule (ca) with a fibrinous cover (fc), containing clusters of large atypical lymphoid cells (square in D, enlarged in **D**-**E**), expressing CD30 (**F**), and B-cell markers CD20 (**G**), OCT2, PAX5 and BOB1, and also the hypoxia marker CA-9 (**H**); Presence of EBV was shown by EBER ISH (**I**), displaying an EBV latency type III, with expression of LMP1 (**J**) and EBNA2 (**K**); There was no T-cell marker expression, but the cells were positive for PD-L1 (**L**)

A 45-year-old female known with a fluctuating swelling of the left breast since 2 years, recently noticed pain in the breast, increase in size and accompanying night sweats. Seven years before presentation, the patient received bilateral textured silicone implants for aesthetic reasons. Two years after breast implantation, the patient was diagnosed with HIV, for which, she has been treated with Abacavir/Lamivudine and Dolutegravir, with undetectable viral load at presentation. MRI revealed, besides intact breast implants, a seroma with intralesional septations medially of the implant at the left breast (see Fig. 2A). PET-CT-scan demonstrated a hypermetabolic lesion in the left breast whereas axillary lymph nodes only showed moderate hypermetabolism. Furthermore, PET-CT also revealed a nodule in the left lung, interpreted of infectious origin. Cytology of the FNAC of the lesion in the left chest showed a mixed infiltrate of neutrophils, lymphocytes, histiocytes, and sparse larger atypical lymphoid cells. These large cells showed immunoreactivity for CD30 (see Fig. 2B) and CD20 whereas the CD3 was only present in small reactive T-lymphocytes. Implants were surgically removed and microscopic examination of the capsulectomy specimen demonstrated no mass formation, but a sclerotic capsule surrounded by a wealthy amount of fibrin. Within this fibrin, a population of medium-sized to large lymphoid cells was found. The infiltrate was confined to the fibrin, with no obvious infiltration into the fibrous capsule (see Fig. 2C-D). The large-sized cells expressed CD30 (see Fig. 2F), but no T-cell markers (CD2-, CD3-, CD4-, CD8-, TIA-, Perforin-, GranzymB-), nor CD15. Furthermore, these cells showed expression of a complete B-cell phenotype (CD20 + , PAX5 + , OCT2 + , CD79A + , CD19 + , BOB.1 +), were of non-germinal center origin (according to the Hans algorithm: CD10-, BCL6 + , MUM1 +), showed a 100% Ki67 proliferation index and uniform expression MYC and PD-L1 (see Fig. 2G and L). There was expression of CA-9 (see Fig. 2H). Malignant cells were negative for HHV8, and positive on EBER ISH expressing an EBV latency type 3 (LMP1 + , EBNA2 +) without lytic activity (BZLF1-) (see Fig. 2I-K). PCR on the peripheral blood indicated only a limited elevation in EBV DNA (< 150 EBV IU/mL). Multiplex PCR-based immunoglobulin (Ig) clonality testing revealed a clonal peak within a polyclonal background for the Ig Kappa target, whereas Ig Heavy Chain was polyclonal. There was no evidence for a MYC rearrangement on FISH and FISH for 9p24 was not interpretable. Bone marrow biopsy was normal, and the axillary glands were not biopsied.

The diagnosis of a breast implant-, fibrin-associated EBV + DLBCL (as part of the spectrum of CI-DLBCL) in an HIV patient was made. Besides surgical removal of both prostheses and capsulectomy, the patient did not receive additional therapy.

During the follow-up period, the patient presented with bilateral tonsillar swelling, but the tonsillar biopsy revealed a reactive EBV-negative follicular hyperplasia. EBV titer in the blood remained negative and HIV-1 RNA viral load was detectable, yet less than 20 copies/mL. After 12 months, follow-up PET-CT showed no evidence for recurrent disease and showed a decrease in volume of the lymph nodes. Clinically the patient is in remission 13 months after diagnosis. Plan is to follow up patient via EBV viral load detection using PCR.

## Discussion and overview of the literature

We report two cases of EBV + DLBCL arising in relation to breast implants. Both lymphomas contained CD30 + large atypical cells, a typical feature of BIA-ALCL, the most common lymphoma type, seen in association with breast implants. BIA-ALCL has recently been recognized a separate entity by the WHO HAEM5 and the ICC, whereas BIA-DLBCL has only been reported sporadically and may therefore be underrecognized [1, 2].

According to recent lymphoma classifications, case 1 was diagnosed as DLBCL-CI in a patient with prior history of chemotherapy and a breast implant. DLBCL-CI arises in immune-privileged sites, in the context of long-standing chronic inflammation, driven by local immunosuppression. Prototype of this disease is pyothorax-associated lymphoma (PAL), but DLBCL-CI also occurs with chronic osteomyelitis, or surgical mesh- or metallic implants. All of the above show strong association to the oncovirus EBV. The immune privileged site and chronic inflammation supports proliferation of EBV-transformed B-cells, that cannot be countered by the host immune response, as evidenced by the EBV latency type 3 [4, 5]. DLBCL-CI is generally associated with mass formation and is considered an aggressive disease [6]. However, our patient could not receive additional therapy and showed complete metabolic remission 6 months post-surgery. Yet, evidence is being accumulated on some cases of DLBCL-CI with an indolent behavior, such as EBV + DLBCL arising in association with cardiac myxomas [7].

In literature, two other cases presented with a mass forming EBV-related BIA-DLBCL [8, 9]. One case did not receive systemic treatment and could unfortunately only be followed up for 2 months. The other patient received chemotherapy R-CHOP, and showed complete metabolic remission but relapsed after one year.

Case 2 fulfilled the criteria for the diagnosis of fibrin-associated DLBCL (FA-DLBCL), often considered a subcategory of DLBCL-CI. This subcategory was introduced after the reports of incidental findings of EBV + large B-cell lymphomas associated with pseudocysts, prosthetic cardiac valves, chronic subdermal hematomas, atrial myxomas and hematomas. These lesions occur in a restricted anatomical space and present as atypical EBV + B-cells in a fibrin background and are related to excellent clinical outcome. Our case 2 did not show mass formation or capsular infiltration and the malignant population was embedded in fibrin. To avoid missing these lymphomas, it is imperative to submit and process also the fibrin clot attached to the breast capsule for histological examination. Rinsing off the capsule can result in loss of the fibrin and tumor cells and is thus strongly discouraged. Capsulectomy was sufficient for the patient to receive complete metabolic remission. In the literature, we found 15 other cases of EBV + BIA-DLBCL without any evidence of mass formation, only one of them was invasive in the capsule [10] (see Table 1).

**Table 1:** Overview of literature reported EBV+ BIA-DLBCL

**Reference**	**Age**	**TimeI->D(yrs)**^**+**^	**Mass formation**	**FA/CI-DLBCL**	**Immunosuppression**	**B-cell Phenotype**	**T-cell phenotype**	**Other IHC markers**	**EBV latency**^**++**^	**Molecular study**	**Staging**^**+++**^	**Treatment**	**Follow-up (months and outcome)**
Morgan S, et al. [11]	69	12	No	NA	Treatment for breast carcinoma (chemotherapy+RT)	CD45^+^, CD20^+^, PAX5^+^, CD79A^+^, CD30^-/+^ (weak), MUM1^+^, BCL6^+^, CD10^-^, CD138^-^	CD45^+^,CD5^-^, CD2^-^, CD3^-^, CD4^-^, CD8^-^, ALK^-^, CD30^-/+^ , Granzyme B^-^, perforin^-^	MYC^+^ (30%), CD138^-^, Ki-67 proliferation index: 80%, HHV8^-^ , AE1/AE3^-^	NA	IGH, IGK clonal gene rearrangement, no TCRb or g clonal rearrangement	T3N0M0 (BM aspirate+biopsy: lymphoma clear)	Capsulectomy	CR (Follow-up 24 months)
Morgan S, et al. [11]	53	9	No	FA-DLBCL	Unknown	CD20^+^, PAX5^+^, CD30^+^, BCL2^+^ (100%), MUM1^+^, BCL6^-^, CD10^-^, CD19^+^, CD79A^+^, OCT2^-^, CD138^-^	CD30^+^, CD5^-^, CD3^-^, ALK^-^, CD2^-^	MYC^+^(50-60%), CD68^-^, HHV8^-^, calretinin^-^, EMA^-^, CAM5.2^-^, MIB-1 proliferation index > 90%	NA	FISH: MYC gene rearrangement without BCL2 gene rearrangement.	T2N0M0	Capsulectomy + 4 cycli R-CHOP	CR (Follow-up 36 months)
Mansy M., et al. [12]	46	5	No	FA-DLBCL	NA	CD30^+^, CD20^+^, PAX5^+^, BCL6^+^, MUM1^+^, CD10^-^BCL2^+^	CD30^+^, ALK^-^, CD2^-^, CD3^-^, ALK^-^	EMA^+^, HHV8^-^, Ki67 proliferation index > 90%	III	clonal rearrangement for the V-J(FR2) target, polyclonal pattern in all IGK targets. polyclonal pattern in TRG targets (TRB not analyzed).	T3N0M0	Capsulectomy	CR (Follow-up 24 months)
Malata M. C., et al. [13]	51	21	No	FA-DLBCL	HIV	CD30^+^, CD45^+^, PAX5^+^, CD79A^+^, BCL2^+^, MUM1^+^, BCL6^+^, CD10^-^, CD138^-^	CD30^+^, CD45^+^, CD3^-^, CD5^-^, CD2^-^, ALK^-^	CD138^-^, HHV8^-^, MIB-1 proliferation index: 80%	NA	NA	T2/3N0M0 Small volume axillary nodes on CT	Capsulectomy	CR (Follow-up 24 months)
Khoo C., et al. [14]	70	9	No	FA-DLBCL	Unknown	CD30^+^, CD45^+^, CD79A^+^, CD20^+^, PAX5^+^, BCL6^+^, MUM1^+^, PD-L1^+^ (SP142 clone), CD10^-^	CD45^+^, CD30^+^, CD3^-^, CD2^-^, CD4^-^, CD5^-^, CD7^-^, CD8^-^, TIA1^-^, perforin^-^, Granzyme B^-^, ALK^-^	CD68^-^, AE1^-^/AE3^-^, Cam 5.2^-^, EMA^-^, HHV-8^-^	NA	Monoclonal peak in IHC rearrangement	T3N0M0	Capsulectomy	CR (Follow-up 12 months)
Mescam L., et al. [10]	72	NA	No	intermediate features between CI- and FA-DLBCL	Unknown	CD30^+^,CD45^+^, CD19^+^, CD20^+^, CD22^+^, CD79A^+^, PAX5^+^, MUM1^+^, CD138^+^, BCL2^+^	CD30^+^,CD45^+^, CD56^-^	CD56^-^, HHV8^-^ Ki67 (100%)	III	Major monoclonal Ig rearrangement and minor TCR rearrangement (1-2% T cells) FISH: no rearrangement of BCL2, BCL6, MYC. tNGS (30% tumor cells): damaging somatic mutations of IRF4 (p.L24F; VAF 10.4%),	T3N0M0 (normal PET scan)	Capsulectomy	CR (19 months after surgery).
Mescam L., et al. [10]	61	NA	No, invasive	intermediate features between CI- and FA-DLBCL	Unknown	CD30^+^, CD45^+^, CD79A^+^, MUM1^+^, CD10^+^, BCL6^+^, Kappa^+^, CD138^+^	CD45^+^, CD4^+^, CD30^+^, CD56^+^	EMA^+^, CD56^+^, CD138^+^, HHV8^-^ Ki67 (100%)	III	Major monoclonal IG Rearrangement and minor TCR rearrangement (10% T-cells) FISH: IGH-MYC translocation, no rearrangement of BCL2 or BCL6.	pT4 (normal PET scan)	Capsulectomy	CR (Follow-up 21 months)
Mescam L., et al. [10]	69	NA	No	intermediate features between CI- and FA-DLBCL	Unknown	CD30^+^, CD45^+^, CD19^+^, CD20^+^, CD22^+^, CD79A^+^, MUM1^+^, BCL6^+^	CD30^+^, CD45^+^, CD56^-^	CD56^-^, CD138^-^ HHV8^-^ Ki67 (80%)	III	Ig gene replacements and a small TCR, FISH: no rearrangement of BCL2, BCL6, MYC.	T3N0M0 (Normal PET scan)	Capsulectomy + 3X R-CHOP	CR (20 months follow-up)
Rodriguez-Pinilla S., et al. [9]	55	15	no	NA	NA	CD30^+^, CD20^+^, CD79A^+^, PAX5^+^, MUM1^+^	CD30^+^, CD3^-^, TIA1^-^, perforin^-^, granzyme B^-^	p53^-^, MYC^-^, HHV8^-^	III	NA	T3N0M0	Capsulectomy	CR (Follow-up 7 months)
Rodriguez-Pinilla S., et al. [9]	59	10	no	NA	Unknown ***	CD30^+^, CD20^+^, CD79A^+^, PAX5^+^, MUM1^+^	CD3^-^, TIA1^-^, perforin^-^, granzyme B^-^, CD30^+^	p53^-^, MYC^-^, HHV8^-^	II or III (LMP1+)	NA	T3N0M0	Capsulectomy	CR (Follow-up 41 months)
Rodriguez-Pinilla S., et al. [9]	63	20	yes	NA	Unknown ***	CD30^+^, CD20^+^, CD79A^+^, PAX5^-^, MUM1^+^	CD3^+^, TIA1^+^, perforin^+^, granzyme B^+^, CD30^+^	p53^-^, MYC^-^, HHV8^-^	III	NA	pT4	Capsulectomy	CR (Follow-up 2 months)
Medeiros J., 2021 [8]	48	21	Yes, invasive	NA	Unknown	CD30^+^, CD45^+^, PAX5^+^, CD79A^+^, CD15^+^, MUM1^+^	CD30^+^, CD45^+^	/	II	NA	pT4 **(**left axillary mass)	Capsulectomy + 4 cycli of R-CHOP	CR + relapse** (Follow-up 71 mo.)
Medeiros J., 2021 [8]*	47-71 (median: 65)	Median10 yrs (4-26)	No	NA	Unknown	CD30 6/7 (85%) CD45 7/7 (100%) CD20 7/7 (100%) PAX5 6/6 (100%) CD79A 7/7 (100%) MUM1 7/7 (100%) BCL6 1/5 (20%) CD10 0/7 (0%) CD138 0/5 (0%)	CD30 6/7 (85%) CD45 7/7 (100%) CD3 0/5 (0%) CD4 0/2 (0%) CD5 0/5 (0%) CD8 0/2 (0%) ALK 0/7 (0%) Granzyme B 0/1 (0%) TIA1 (0/1 (0%)	Myc 5/6 (83%) Ki-67 proliferation median 80% EMA 0/2 (0%) HHV-8 0/7 (0%)	III	IGH 3/3 (100%)	pT2 7/7 (100%)	6/7 Capsulectomy 1/7 Capsulectomy + 3X R-CHOP	CR (Follow-up median: 8 (1-96) months)
Case 1	75	28	Yes	CI-DLBCL	Treatment for breast carcinoma (chemotherapy) + hypoglobulinemia	CD30^+^, CD20^+^, CD79A^+^, BOB1^-^, PAX5^+^, OCT2^+^, CD15^+^, CD19^-^, MUM1^+^, CD10^-^, BCL6^+^	CD30^+^, CD3^-^, CD4^-^, CD8^-^	Ki67 (70-80%), BCL2^+^, C-MYC (30%), PD-L1^+^	III	FISH gain of region 9p24	pT4pR0	Capsulectomy	CR (after 6 months)
Case 2	45	8	No	FA-DLBCL	HIV	CD30^+^ , CD20^+^, PAX5^+^, OCT2^+^, BOB1^+^, CD79A^+^, CD10^-^, CD15^-^, BCL6^+^, MUM1^+^, GATA3^+^	CD30^+^, CD2^-^, CD3^-^, CD4^-^, CD8^-^, CD5^-^, TIA1^-^, Perforin^-^, Granzyme B^-^	C-MYC in ~75% of atypical cells, BCL2^+^, Ki67 (>95%), HHV8^-^, PD-L1^+^	III	IgH polyclonal, IgKA monoclonal in polyclonal background, IgKB polyclonal	pT2N0M0 (Bone marrow lymphoma clear)	Capsulectomy	CR (Follow-up 12 mo)

^+^ Time I->D: from implant to diagnosis

^++^ EBV latency patterns by immunohistochemmistry: 0/I = LMP1- and EBNA2-; II = LMP1+ and EBNA2-; latency III = LMP1+ and EBNA2+

^+++^ Following staging guidelines described in Clemens *et al.,* 2019 [3]

* Review of 7 non-invasive EBV+ BIA-DLBCL including 2 cases from Khoo C., et al. [14] and Mescam L., et al. [10], also shown in this table

** relapse in the ipsilateral axilla 24 months after chemotherapy for which patient received chemo-and radiotherapy, after which, patient was in complete remission. Subsequently, the patient had a second lymphoma relapse presenting as a chest wall mass 21 months after chemotherapy, for which she received Chemotherapy. The patient achieved complete remission and received an autologous stem cell transplant (ASCT). She is currently in complete remission 8 months after ASCT and 71 months from the initial diagnosis

*** History of breast cancer but no information on treatment in report

Four reports refer to their case as FA-DLBCL whereas Mescam et al., refer to their cases as having intermediate features between FA-DLBCL and CI-DLBCL [10]. Three cases were incidental findings, a feature that is typical of FA-DLBCL, whereas all other described cases in the literature were symptomatic. Medeiros J., et al. [8] tried to track down the etiology of BIA-DLBCL by comparing the disease with BIA-ALCL and FA-DLBCL. Similarities with FA-DLBCL included the absence of mass-formation, the often incidental finding and the fibrinous background. On the other hand, the long latency interval (21 years) between implantation and lymphoma formation is more similar to BIA-ALCL, as is the morphological appearance [8].

In order to understand the clinical-pathological characteristics of BIA-DLBCL, we reviewed the available data from 19 reported EBV + BIA-DLBCL cases so far (see Table 1). BIA-DLBCL develops after a median time of 10–15 years post breast implant, and at a median age of 62.5 years old (8–14). It either presents asymptomatically (16%) or with discomfort (68%) (breast swelling or pain), while only in 3 cases there was palpable nodularity or a rapidly growing mass. B-symptoms were restricted to weight loss in case 1 and night sweats in case 2 of our reported cases, no other B-symptoms were reported in the reviewed cases. Immunophenotypical characteristics include the expression of CD30 in 18/19 cases (95%) and expression of CD45 in 12/13 (92%) of the reported cases. B-cell marker expression is largely retained with expression of CD20 (100%, 16/16), PAX5 (94%, 16/17) and CD79A (100%, 18/18). Nearly all lymphomas were of non-GCB phenotype (absence of CD10 in 13/14 cases, BCL6 + in 7/12 cases, MUM1 + in 19/19 cases). Aberrant T-cell marker expression in the neoplastic B-cells, such as CD3 and CD4, occurred only occasionally, in 1/14 and 1/6 cases, respectively. Testing for other T-cell marker expression, such as CD2, CD5 and CD8 was absent in 5, 10 and 5 patient samples, respectively. Proliferation index was high (Ki67 > 70%) in all tested cases (15/15) and C-MYC was positive in 7/11 cases. In the 3 cases tested, PD-L1 was expressed in the tumor cells, which is indicative of local immune suppression. Furthermore, in all cases, in which EBV latency was checked, almost all of them (9/10, 90%) showed a latency type 3 (see Table 1).

In post-transplant lymphoproliferative disorders (PTLD) or immune modulatory agent-related lymphoproliferative disorders (IAR-LPD), EBV latency type 3 often observes and indicates an abrogated immune system and the lack of adequate immune response. In a breast implant setting, where occasionally lymphomas arise in the capsule, an infiltrating tumor microenvironment is generally lacking, which suggests an immune-privileged site for EBV-transformed B-cells to survive. This can be the leading cause of EBV-related transformation of B-cells. Detection of latency type 3 should always warrant the clinician to look for potentially correctable immune dysregulations. Both of our presented cases have a degree of immune dysregulation, related to the prior chemotherapy-regimen, hypogammaglobulinemia and HIV infection (although under control at the time of lymphoma diagnosis). So, EBV reactivation in an immune-privileged site, as supported by PD-L1 upregulation indicating immune escape, might play a role in the pathogenesis of EBV + BIA-DLBCL, possibly assisted by genetic aberrations as were found in MPS results in lymphoma associated genes DDX3X, EZH2 and NFKBIE of the mass-forming lesion in Case 1 [15, 16]. Although BIA-ALCL is not associated with EBV, Oishi et al*.,* also suggested a peri-implant tumor microenvironment (TME) as potential attributing factor in the pathogenesis of BIA-ALCL and they have also proven that this TME is hypoxic. RNA-sequencing revealed that the hypoxia marker CA-9 was significantly upregulated in BIA-ALCL compared to non-BIA-ALCL [17]. In another case-report on an unusual presentation of BIA-ALCL with no mass formation or capsular infiltration but with lymph adenopathy, CA-9 was expressed in neoplastic cells of primary tumor and in the lymph node but not in areas of diffuse lymph node involvement which is why they suggested that the hypoxic program could be involved early after metastasis but disappear in more extensive spread and vascularization [18]. Our case 2, with DLBCL in a fibrinous background, also showed CA-9 expression, indicating an hypoxic environment, whereas the mass forming Case 1 showed no expression of CA-9, which is in line with the scarce reports on CA-9 in BIA-ALCL [17, 18]. Yet, we encourage further investigation of CA-9, as possible biomarker and its (potential driver) role in pathogenesis.

It is not clear how to stage BIA-DLBCL. BIA-ALCL guidelines follow TNM staging, in which pT4 is reported to be associated with adverse prognosis [19]. However, our reported case 1 displayed a pT4N0 (according to the ALCL TNM guidelines), and performed well more than one year after surgical removal. Applying the ALCL TNM guidelines in the reported BIA-DLBCL cases (see Table 1), 7/19 (37%) corresponded to pT2, for 1/19 cases, it was not clear if it corresponded to pT2 or pT3, 7/19 (37%) cases corresponded to pT3 and 4/19 (21%) to pT4, of which three cases showed mass formation. Only one of the four cases with pT4 (and mass formation), had a more aggressive disease course, with relapse 24 months after capsulectomy and 4 cycles of R-CHOP [8]. All other reported cases showed complete response, either after capsulectomy alone in 16/19 (84%) or capsulectomy and chemotherapy (3–4 cycli of R-CHOP) in 3/19 cases (16%). However, some reservations have to be made due to the short time of follow-up in some cases (between 1–96 months, median of 19 months follow-up time). In BIA-ALCL, the rate of disease events after surgical removal of a T4 disease is 14,3% whereas in patients with T1-2 stage disease, this was 0% [20]. In a report of Boyer et al*.* on 47 FA-DLBCL cases, there was only 2% (1/47) disease-related death and 6.4% (3/47) of persistent or recurrent disease [6]. As a comparison a review of 267 CI-DLBCL cases by the same authors showed 54% (144/267) disease-related deaths [6]. So far, no disease-related mortalities were reported in any of the 19 reported EBV + BIA-DLBCLs.

Here, we report 2 cases of localized EBV + BIA-DLBCL with indolent behaviour, even in the case of mass formation, suggesting a different, less aggressive clinical course than CI-DLBCL, type PAL [1]. However, further studies are needed to support the hypothesis that a conservative approach is advisable in BIA-DLBCL.

## Conclusion

We report two cases of EBV + DLBCL related to breast implants, either presenting as a fibrin-associated DLBCL or a mass-forming CI-DLBCL. They illustrate that the detection of large atypical CD30 positive cells in this setting is not sufficient for the diagnosis of BIA-ALCL, and that BIA-DLBCL can be overlooked if the cell of origin is not further determined. Increased awareness for this entity is needed, and we recommend an extended panel of B- and T-cell immunohistochemical markers and EBV in situ hybridization.

Whether BIA-DLBCL constitutes a separate clinicopathological entity or should reside under the CI-DLBCL or FA-DLBCL category remains to be determined.

## Data Availability

All data generated or analyzed during this study is available from the corresponding author upon request.
